# Identification of PANoptosis-relevant subgroups to evaluate the prognosis and immune landscape of patients with liver hepatocellular carcinoma

**DOI:** 10.3389/fcell.2023.1210456

**Published:** 2023-05-30

**Authors:** Zhengwei Zhang, Feng Zhang, Ping Pang, Yapeng Li, Xiaoning Chen, Shibo Sun, Yu Bian

**Affiliations:** ^1^ Department of Pharmacology (The State-Province Key Laboratories of Biomedicine-Pharmaceutics of China, Key Laboratory of Cardiovascular Research, Ministry of Education), College of Pharmacy, Harbin Medical University, Harbin, China; ^2^ The Second Affiliated Hospital of Harbin Medical University, Harbin, China

**Keywords:** PANoptosis, liver hepatocellular carcinoma, prognosis, immunotherapy, programmed cell death

## Abstract

Liver hepatocellular carcinoma (LIHC) is one of the most common malignant tumors, which is difficult to be diagnosed at an early stage due to its poor prognosis. Despite the fact that PANoptosis is important in the occurrence and development of tumors, no bioinformatic explanation related to PANoptosis in LIHC can be found. A bioinformatics analysis on the data of LIHC patients in TCGA database was carried out on the basis of previously identified PANoptosis-related genes (PRGs). LIHC patients were divided into two PRG clusters whose gene characteristics of differentially expressed genes (DEGs) were discussed. According to DEGs, the patients were further divided into two DEG clusters, and prognostic-related DEGs (PRDEGs) were applied to risk score calculation, the latter of which turned out to be practical in identifying the relationship among risk score, patient prognosis, and immune landscape. The results suggested that PRGs and relevant clusters were bound up with the survival and immunity of patients. Moreover, the prognostic value based on two PRDEGs was evaluated, the risk scoring model was constructed, and the nomogram model for predicting the survival rate of patients was further developed. Therefore, it was found that the prognosis of the high-risk subgroup was poor. Additionally, three factors, namely, the abundance of immune cells, the expression of immune checkpoints, and immunotherapy and chemotherapy were considered to be associated with the risk score. RT-qPCR results indicated higher positive expression of CD8A and CXCL6 in both LIHC tissues and most human liver cancer cell lines. In summary, the results suggested that PANoptosis was bound up with LIHC-related survival and immunity. Two PRDEGs were identified as potential markers. Thus, the understanding of PANoptosis in LIHC was enriched, with some strategies provided for the clinical therapy of LIHC.

## Introduction

Hepatic carcinoma is the sixth most common cancer and the fourth most frequent cause of cancer mortality throughout the world. Although liver hepatocellular carcinoma (LIHC) and intrahepatic cholangiocarcinoma (iCCA) are two typical forms of primary liver cancer, the incidence rate of LIHC accounts for 70% (Bray et al., 2018; Beaufrère et al., 2021). With the wide vaccination against hepatitis B and the application of antiviral drugs, the proportion of viral LIHC has decreased, and thus nonalcoholic steatohepatitis (NASH) has been considered to be an important cause of LIHC (Karim et al., 2022). In the past 10 years, several treatment methods, including surgical therapy and nonsurgical therapy, have been proven to have positive effects on liver cancer. However, the high malignancy and occult nature of LIHC left most patients with advanced or terminal cancer few opportunities for surgical eradication (Chung et al., 2022). In recent years, the treatment of LIHC has entered the era of immunotherapy. The experimental results from CheckMate 040 and Keynote-224 pointed out that two programmed death 1 (PD-1) inhibitors (nivolumab and pembrolizumab) were approved as second-line therapy for LIHC by the U.S. Food and Drug Administration (El-Khoueiry et al., 2017; Zhu et al., 2018).

To maintain the physiological balance under normal or stressful conditions, cells undergo a series of cell death pathways. Of all recognized types of programmed cell death (PCD), pyroptosis, apoptosis, and necroptosis are typical ways to control the initiation, transduction, and execution of cell death through complicated molecular mechanisms (Galluzzi et al., 2018; Kesavardhana et al., 2020). The biological functions of three PCDs have been discussed in previous studies, respectively, but they were found not to exist separately in subsequent studies. Research studies have been conducted on common biomarkers from three PCDs in injury or infectious diseases (Malireddi et al., 2020a; Banoth et al., 2020; Karki et al., 2020; Zheng et al., 2020; Karki et al., 2021a). The following question arises: are the three modes triggered individually or taken control by a sort of cell death induction compound, PANoptosome, that has been identified? The pathway in which the compound is involved has been named PANoptosis (Wang and Kanneganti, 2021). Through the regulation of PCD pathways, it is noted that the majority of chemotherapy drugs have reached a presentable therapeutic effect. However, owing to mutations in tumor cells, drug tolerance can often be seen in PCD pathways, but with stimulated PANoptosis triggering immune system activation and drug-resistance reduction (Malireddi et al., 2020b). For example, PANoptosis could be induced in a variety of cancer cell lines by IFNγ in combination with TNFα, which diminished the size of tumors in immune-deficient mice (Malireddi et al., 2021). Additionally, oncogenesis was promoted with PANoptosis inhibited after interdicting the interplay of ZBP1 and RIPK3 (Karki et al., 2021b). Despite controversies and complexities, the functions of caspase-8 enabled it to become a new target and research hotspot in oncology research and possibly a molecular switch among three cell death pathways (Newton et al., 2019). Hence, it is evident that obtaining more cognition about the impact of PANoptosis on tumors is crucial for exploring novel therapeutic strategies.

At present, in order to predict the survival and immune landscape of cancer patients, various gene sets are employed to construct cancer classification and prognostic features. For instance, Chen et al. (2022a) forecasted the survival prognosis, immune infiltration, and drug efficacy of hepatocellular carcinoma based on pyroptosis-related genes. Among 14 differentially expressed apoptotic genes, Zhu et al. (2020) selected two genes that could effectively predict the diagnosis and prognosis of LIHC. By analyzing necroptosis-related genes, Chen et al. (2022b) discovered novel approaches for risk stratification and LIHC treatment optimization. Nevertheless, there are no data assessing the impact of genes associated with PANoptosis on LIHC from the bioinformatics aspect.

Our latest data suggested that molecular aggregation and prognostic characteristics found in PANoptosis could forecast immunologic and prognostic conditions of LIHC patients. To begin with, 377 LIHC patients were divided into two separate clusters depending on the PANoptosis-related gene (PRGs) expression level. Following the separation, the differentially expressed genes (DEGs) of the aforementioned two clusters assisted in the further division of the patients into additional two clusters. Meanwhile, to predict overall survival (OS) and analyze the effects of immunotherapy in LIHC patients, prognostic characteristics and risk scores were conducted.

## Materials and methods

### Data collection

Based on clinical data and transcriptome datasets of LIHC patients from TCGA (https://portal.gdc.cancer.gov), 374 LIHC and 50 adjacent normal samples were analyzed. A total of 377 cases with complete clinical and pathological information were selected for the study. Definite clinical information of LIHC patients is provided in Supplementary Table S1. R software (version 4.1.1) was used to convert fragments per kilobase million (FPKM) from LIHC of TCGA into transcripts per million (TPM). According to previous research (Malireddi et al., 2019; Malireddi et al., 2020b; Karki et al., 2020; Samir et al., 2020; Briard et al., 2021; Jiang et al., 2021; Lee et al., 2021; Place et al., 2021; Nguyen and Kanneganti, 2022), 19 PRGs were determined, and the details are listed in Supplementary Table S2.

### PRG consensus clustering analysis

The ConsensusClusterPlus package in R language was applied to explore the relationship between LIHC subtypes and PRG expression. We used PAM algorithm for clustering analysis and selected the Euclidean distance as the method for calculating distance. The seed was set to 123,456. The Kaplan–Meier (KM) method and logarithmic rank test were used to contrast prognostic factors of the two clusters; meanwhile, the principal component analysis (PCA) was carried out. The limma package and Wilcoxon test were used to explore the DEGs and clinical characteristic differences derived from two clusters. DEGs were selected with the threshold of |log fold change (FC)| > 2 and *p*-value <0.05. The gene set variation analysis (GSVA) R package was utilized to analyze discrepancy in the biological process. Immune cell infiltration score was counted, and immunological competence was assessed by single-sample gene set enrichment analysis (ssGSEA).

### Functional enrichment analysis of DEGs

Kyoto Encyclopedia of Genes and Genomes (KEGG) and Gene Ontology (GO) analyses were performed to predict the metabolic pathways and gene function, respectively, using ggplot2, Bioconductor, Hs.eg.db, and org R packages. The threshold was set at *p*-value <0.05.

### Establishment of prognostic features based on PANoptosis

Genes used to build the prognostic features based on PANoptosis were identified according to the aforementioned DEGs. Additional two clusters of patients were constructed with genes derived from DEG expression, which were compared on PRG expression, clinicopathological characteristics, and length of survival at once. In addition, the survival, survminer, and glmnet R packages were used to carry out least absolute shrinkage and selection operator (LASSO) regression analyses and multivariable Cox regression analyses. The following formula was used to calculate the individual risk score: $\sum_{i=1}^{n}\beta i*\lambda i$ , where n, βi , and λi represent the number of genes, regression coefficient, and gene expression value, respectively. As a result, two genes were added to the establishment of prognostic features. High-risk and low-risk patients were separated through a median risk score that was estimated depending on both DEG and PRG expression. The discrepancy between the survival time of high-risk and low-risk patients was compared through Kaplan–Meier (KM) analysis, and the predictive accuracy of the model was assessed through performing the area under the curve (AUC) of the receiver operating characteristic (ROC) curve. The nomogram of LIHC patients was constructed according to the risk model, which was checked using a calibration chart. In addition, TCGA data were randomly divided into a training queue and verification queue. In order to avoid the impact of random allocation deviation on the stability of subsequent modeling, all samples were returned to the random grouping 100 times in advance, and the packet sampling was carried out according to the 7:3 ratio of the training queue and the verification queue. According to the median risk score of the training queue and the validation queue, the samples were all divided into high-risk and low-risk subgroups. The KM and ROC analyses were performed for the two queues. Finally, we validated the survival analysis of key genes through the GSE10186 dataset.

### Assessment of the tumor microenvironment in high-risk and low-risk subgroups

Quantity peaks of infiltrated immunocytes, which were obtained from high-risk and low-risk subgroups, were calculated using CIBERSORT, to grasp the relevance to the risk model and tumor microenvironment (TME). Simultaneously, the analysis was performed to identify the relationship of two prognostic genes and the quantity peaks of infiltrated immunocytes. In addition, the Wilcoxon rank test was applied to TME scores consisting of stromal, immune, and ESTIMATE scores between the aforementioned two subgroups. We used the following R packages: “ggplot2” for creating visualizations, “limma” for the analysis of gene expression data, and “plyr” for data manipulation.

### Assessment of mutations, effects on immune therapy, and chemotherapeutics in high-risk and low-risk subgroups

Mutation annotation format (MAF) was programmed through the maftools R package, to grasp gene mutations in LIHC patients from two subgroups. Spearman’s method was utilized to discuss the relationship between the risk model and tumor mutation burden (TMB) score. The tumor immune dysfunction and exclusion (TIDE http://tide.dfci.harvard.edu/) was used to predict the potential immune checkpoint-blocking reaction in LIHC. A comparison between immunologic checkpoint expression and drug IC_50_ in both risk subgroups was conducted, to understand the relationship between the risk model and the effects of immune therapy and chemical agents.

### Evaluation of CD8A and CXCL6 by RT-qPCR

A total of 11 pairs of LIHC and adjacent normal samples were obtained from The Second Affiliated Hospital of Harbin Medical University. Furthermore, this study was approved by the Ethics Committee of the hospital. Meanwhile, expression levels of CD8A and CXCL6 were assessed in human hepatoma cell lines (HepG2, Hep3B, Huh7, HCCLM3, and PLC/PRF/5) and normal human hepatic cell lines (LO2, Chang liver, and WRL68), which were obtained from Zhongqiaoxinzhou Biotech (Shanghai, China). HepG2, Hep3B, Huh7, HCCLM3, Chang liver, and WRL68 were cultured in Dulbecco’s modified Eagle’s medium (DMEM, Gibco Thermo Fisher Scientific, Inc. United States) containing 10% fetal bovine serum (FBS, Biological Industries, Beit Haemek, Israel) and 1% penicillin–streptomycin (P/S, Solarbio, Beijing, China). LO2 was cultured in Roswell Park Memorial Institute 1640 medium (RPMI-1640, HyClone, UT, United States), supplemented with the aforementioned ingredients. In addition, PLC/PRF/5 was cultured in minimum essential medium (MEM). Total RNA was harvested from tissues and cell lines utilizing TRIzol reagent (Invitrogen, United States). cDNA was generated using the synthesis kit (Accurate Biology), and RT-qPCR was carried out utilizing the real-time SYBR Green Premix Pro Taq HS qPCR Kit (code AG11701; Accurate Biology). The primers used for RT-qPCR are listed in Supplementary Table S3.

### Statistical analysis

R version 4.1.0 software was used to conduct the statistical analysis, and |log fold change (FC)| > 2 and *p*-value <0.05 were considered statistically significant.

## Results

### Hereditary variability of PRGs in LIHC

**FIGURE 1:**
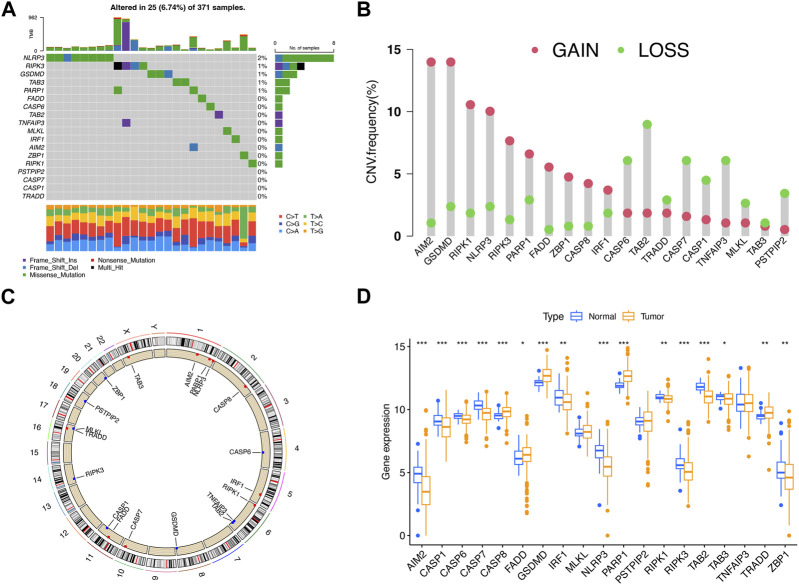
Hereditary variability of PRGs in LIHC. **(A)** Mutation situations of 19 PRGs in LIHC patients. **(B)** Copy number alterations of PRGs. **(C)** Locations of CNV alterations for PRGs on 23 chromosomes. **(D)** Expression difference of PRGs between LIHC and adjacent normal samples. *p < 0.05; **p < 0.01; ***p < 0.001.

To reveal the hereditary variability and differential expression of PRGs, the analysis of 377 LIHC patients was performed, which included the comparison of 374 LIHC and 50 adjacent normal samples. The investigation of the somatic mutagenesis rate of 19 PRGs in LIHC patients revealed that 25 PRGs (6.74%) from 371 samples showed a mutation, and the maximum mutation frequency was observed for NLRP3 (Figure 1A). The positions of copy number variation (CNV) changes for PRGs were observed on human chromosomes (Figure 1B). The maximum CNV was observed for AIM2, GSDMD, PIPK1, and NLRP3, whereas there was a diminished CNV for CASP6, TAB2, CASP7, and TNFAIP3 (Figure 1C). There existed differential expression of 16 PRGs between LIHC and adjacent normal samples, among which the expression of CASP8, FADD, GSDMD, PARP1, and TRADD was upregulated, whereas that of AIM2, CASP1, CASP6, CASP7, IRF1, NLRP3, RIPK1, RIPK3, TAB2, TAB3, and ZBP1 was downregulated in tumor samples (*p* < 0.05) (Figure 1D).

### Construction of PRG clusters in LIHC

**FIGURE 2:**
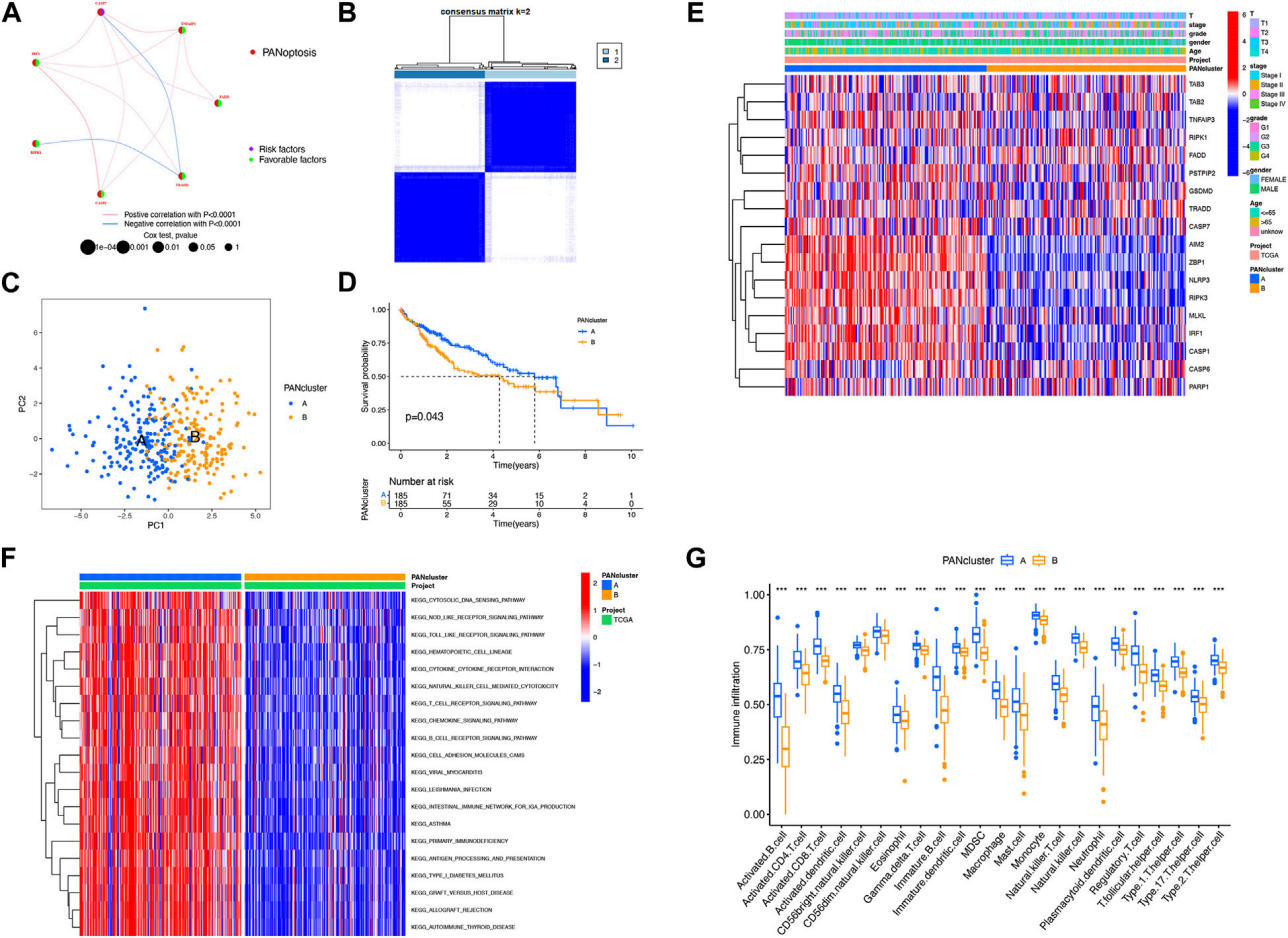
Construction of PRG clusters in LIHC. **(A)** Interactions among PRGs in LIHC. **(B)** Construction of two PRG clusters depending on consensus clustering analysis. **(C)** Good distribution between two PRG clusters shown on PCA. **(D)** Better prognosis from PRG cluster A (*p* = 0.043). **(E)** Relationship among PRG clusters, clinical features, and PRG expression shown on the heat map. **(F)** Difference in the enriched pathways between PRG clusters. **(G)** Difference in immune cell infiltration between PRG clusters. ***p < 0.001.

To explore the relationship between LIHC subtypes and PRG expression, PRG clusters were constructed. The interplay and prognostic effects of PRGs were illustrated in the net graph (Figure 2A). Consensus clustering analysis was performed to determine clusters with the best interclass relevance and the worst intergroup relevance, which discussed the relationship between LIHC classification and PRG expression. To study what situations satisfied the criterion requirement, we adjusted the cluster variables (k) and derived at k = 2 (Figure 2B; Supplementary Figure S1). Two PRG clusters of LIHC patients were classified satisfactorily (Figure 2C). We added the KM curves, which revealed an association between LIHC prognosis and PRG expression (Supplementary Figure S2). In addition, there was a statistical difference in survival duration between PRG clusters A and B (*p* = 0.043) (Figure 2D). A heat map was constructed to explore the relationship among clinical characteristics, PRG expression, and PRG clusters (Figure 2E). Compared with PRG cluster B, immune-associated pathways accumulated in PRG cluster A, such as NOD-like and toll-like receptor signaling pathways and T-cell receptor signaling pathway (Figure 2F). ssGSEA was performed to compare infiltration of immunocytes between two clusters, the outcome of which demonstrated that activated B cells, activated CD4^+^/CD8^+^ T cells, and others showed heightened levels in PRG cluster A (Figure 2G). In conclusion, PRGs were related to prognosis and immunity in LIHC.

### Identification of DEGs in PRG clusters

**FIGURE 3:**
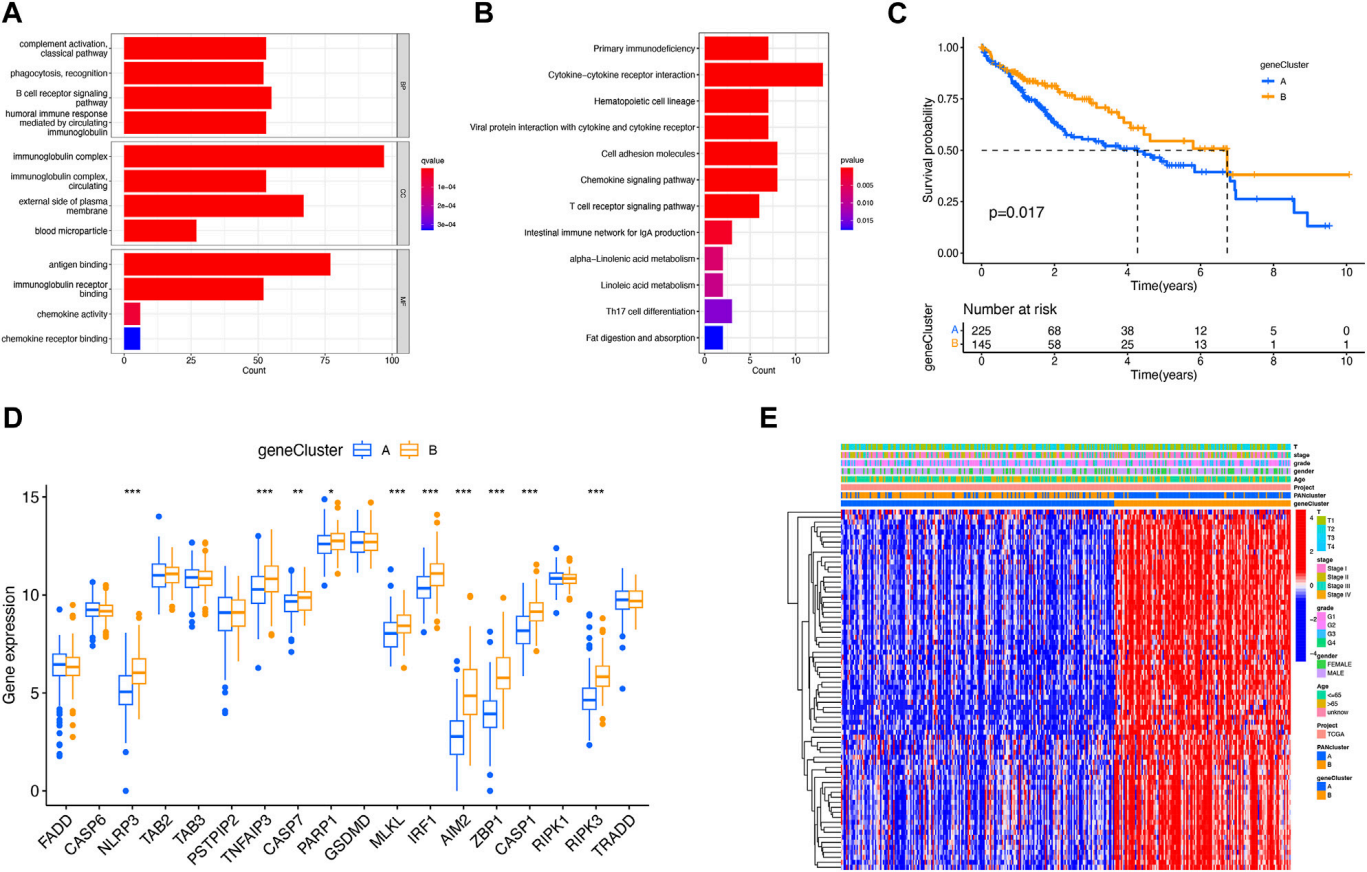
Identification of DEGs for PRG clusters. **(A)** GO analysis of DEGs. **(B)** KEGG analysis of DEGs. **(C)** Better prognosis from DEG cluster B (*p* = 0.017). **(D)** Expression difference of PRGs between two DEG clusters. **(E)** Relevance between DEG clusters and clinical features shown on the heat map. *p < 0.05; **p < 0.01; and ***p < 0.001.

To further explore the relationship between LIHC and PRGs, GO classification and enrichment analysis were performed on the basis of DEGs. These DEGs were involved in biological processes of complement activation and phagocytosis recognition, cellular components of immunoglobulin complex, and molecular functions of antigen binding and immunoglobulin receptor binding (Figure 3A). KEGG analysis demonstrated that DEGs were involved in the cytokine–cytokine receptor interaction pathway, chemokine signaling pathway, and T-cell receptor signaling pathways (Figure 3B). Next, additional two clusters of patients were constructed with genes derived from DEG expression (Supplementary Figure S3). In addition, there was a statistical difference in survival duration between DEG clusters A and B (*p* = 0.017) (Figure 3C). There existed differential expression of 10 PRGs between two DEG clusters, among which the expression of NLRP3, TNFAIP3, CASP7, PARP1, MLKL, IRF1, AIM2, ZBP1, CASP1, and RIPK3 was upregulated in DEG cluster B (Figure 3D). A heat map was constructed to explore the relevance among clinical characteristics, PRG clusters, DEG clusters, and DEG expression (Figure 3E).

### Analysis of prognosis of PANoptosis

**FIGURE 4:**
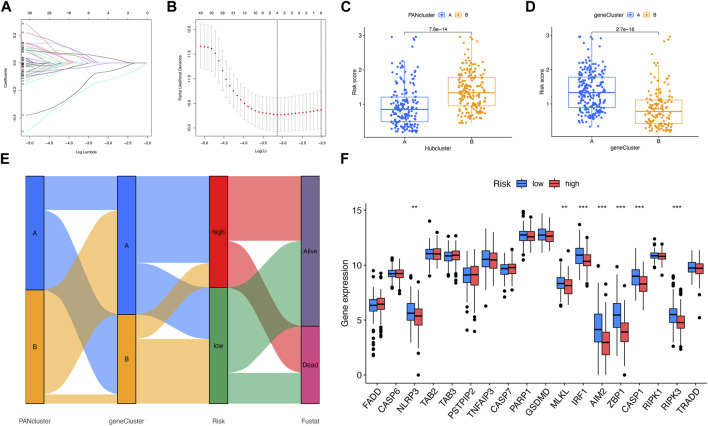
Analysis of risk score. **(A, B)** LASSO regression analysis and partial likelihood deviance on the prognostic genes. **(C, D)** Relevance between risk score and PANoptosis-relevant subgroups. **(E)** Relevance among PANoptosis-relevant subgroups, risk subgroups, and survival status shown on the Sankey plot. **(F)** Expression difference of PRGs in two risk subgroups. **p < 0.01; ***p < 0.001.

**FIGURE 5:**
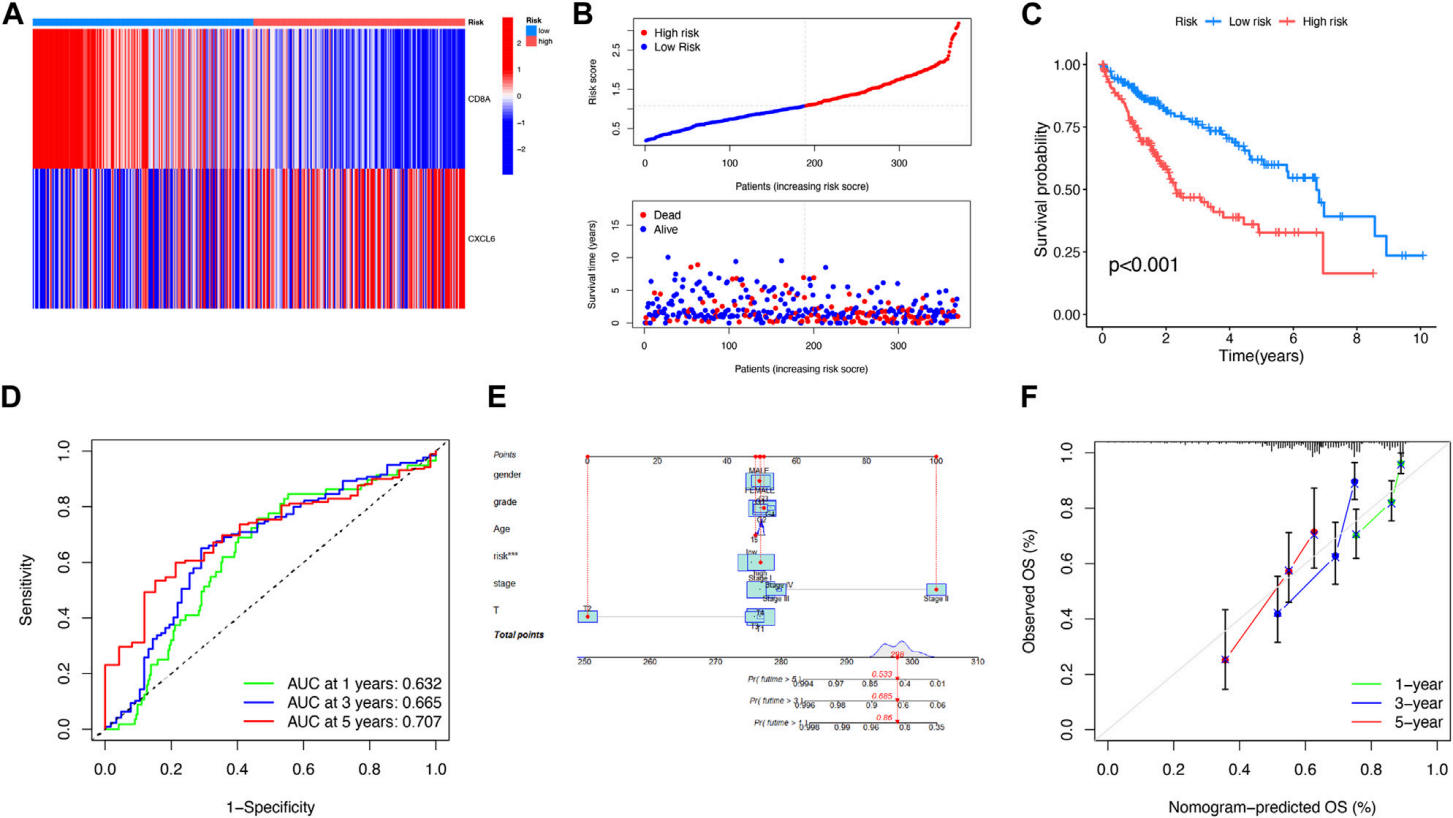
Analysis of the prognosis of PANoptosis. **(A)** Expression of two genes in two risk subgroups shown on the heat map. **(B)** Risk score and survival outcome of each patient. **(C)** Better prognosis from the low-risk subgroup (*p* < 0.001). **(D)** AUCs of 1-, 3-, and 5-year survival periods were 0.632, 0.665, and 0.707, respectively. **(E)** A nomogram using risk score and other clinical features was constructed for predicting the survival of LIHC patients. **(F)** Calibration graphs showed that the actual survival rates of LIHC patients were close to the nomogram-predicted survival rates.

The prognosis-related DEGs (PRDEGs) were selected on the basis of LASSO and Cox regression analyses (Figures 4A, B). The following formula was used relying on two PRDEGs: Risk score = $\sum_{i=1}^{n}\beta i*\lambda i$ , where n, βi , and λi represent the number of genes, regression coefficient, and gene expression value, respectively. The results of the risk score pointed out that PRG cluster A and DEG cluster B were, respectively, lower than their counterparts (Figures 4C, D). The relevance of clusters of PRGs and DEGs to the status of risk and survival was analyzed as shown in Figure 4E. The expression of NLRP3, MLKL, IRF1, AIM2, ZBP1, CASP1, and RIPK3 was higher in the low-risk subgroup (Figure 4F). The relationship between PRDEG expression and risk subgroups was shown in the heat map (Figure 5A). According to the risk score of each patient, patients were divided into low-risk and high-risk subgroups, which showed better survival prognosis of patients in the low-risk subgroup (Figure 5B). Once again, the analysis results of the KM curve supported our view (Figure 5C). Time-dependent ROC curve analysis showed that the AUCs of 1-, 3-, and 5-year survival periods were 0.632, 0.665, and 0.707, respectively, indicating that the risk model had a high predictive ability for prognosis (Figure 5D). The clinical characteristics and risk score were analyzed by the nomogram to predict the 1-, 3-, and 5-year survival of patients with LIHC (Figure 5E). The calibration chart showed that the predicted probability of survival from the nomogram was highly consistent with the observed survival probability (Figure 5F). TCGA data were randomly divided into a training queue and a test queue. The results of KM survival analysis and ROC curve analysis of both queues are shown in Supplementary Figure S4. In the training queue, the survival probability of patients in the high-risk subgroup was significantly lower than that in the low-risk subgroup (*p* < 0.001), and the AUCs of 1, 3, and 5 years were 0.693, 0.704, and 0.750, respectively. Similarly, ideal results were obtained in the test queue, which indicated that risk scores could effectively forecast the patient outcome. In short, the creation of risk score would be helpful for the analysis of prognosis and treatment. In the validation dataset, the results showed that CXCL6, with a *p*-value of 0.042, was a prognostic factor for hepatocellular carcinoma. CD8A had a *p*-value of 0.057 (Supplementary Figure S5).

### Assessment of tumor microenvironment between two risk subgroups

**FIGURE 6:**
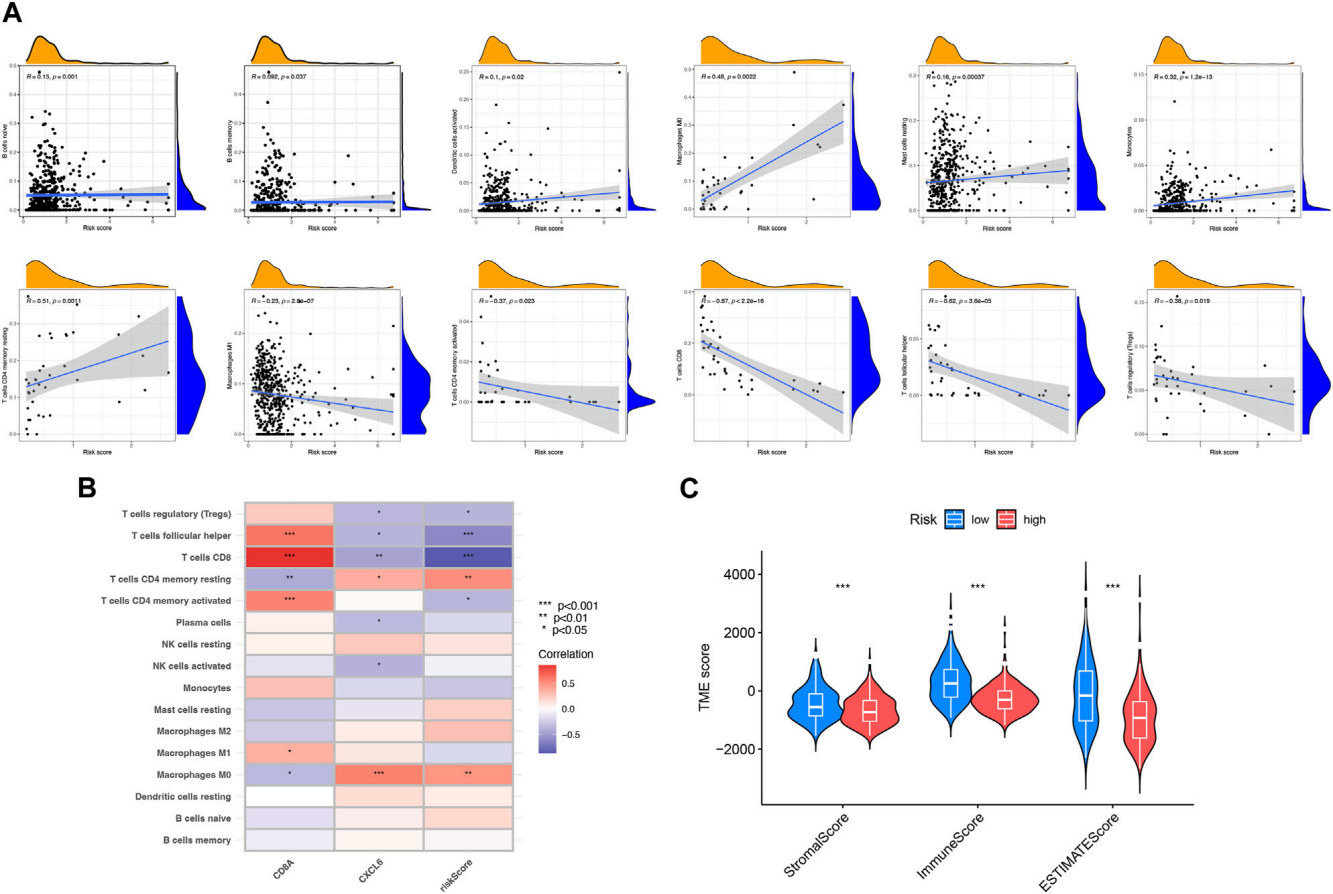
Assessment of the tumor microenvironment between two risk subgroups. **(A)** Relevance between risk score and different immune cell types. **(B)** Relevance between immune cells and two PRDEGs. **(C)** Relevance between risk score and TME scores. *p < 0.05; **p < 0.01; ***p < 0.001.

As illustrated in Figure 6A, the risk score was related to the abundance of immune cells. For instance, naive B cells, memory B cells, activated dendritic cells, and M0 macrophages all had a positive correlation with risk scores. Nevertheless, M1 macrophages activated memory CD4^+^ T cells, CD8^+^ T cells, and follicular helper T cells, where all had a negative correlation with risk scores. The relationship between two PRDEGs and the abundance of immune cells was discussed (Figure 6B). The result showed that the stromal and immune scores of the low-risk subgroup were higher (Figure 6C).

### Assessment of mutation, effects on immune therapy, and chemotherapeutics in two risk subgroups

**FIGURE 7:**
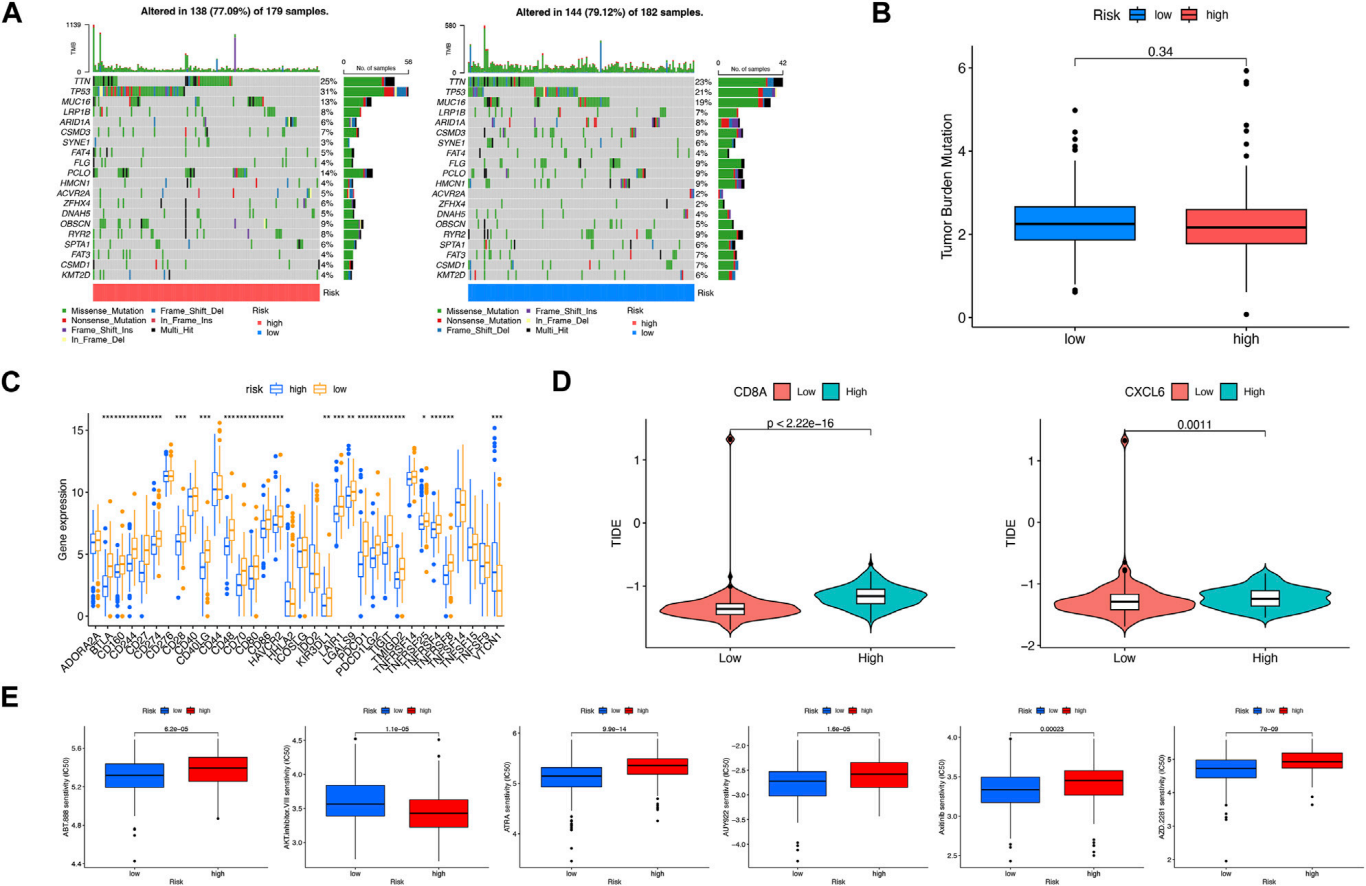
Assessment of mutation, effects on immune therapy, and chemotherapeutics in high-risk and low-risk subgroups. **(A)** Somatic gene mutations in high-risk and low-risk subgroups. **(B)** No significant relevance between TMB score and risk score. **(C)** Differences in immune checkpoint gene expression in high-risk and low-risk subgroups. **(D)** Relevance between risk score and TIDE score. **(E)** Significant IC_50_ difference from six therapeutic drugs between two risk subgroups. *p < 0.05; **p < 0.01; ***p < 0.001.

To explore the discrepancy of gene mutations between two risk subgroups of LIHC patients, the maftools R package was adopted, which showed TTN, TP53, MUC16, LRP1B, and ARID1A had the most mutations in both risk subgroups (Figure 7A). In addition, TMB had no significant associations with risk scores (Figure 7B). There were many immune checkpoint genes existing differences in expression between two risk subgroups, which included BTLA, CD160, CD244, CD274 (PD-L1), and PDCD1, with high expression in the low-risk subgroup (Figure 7C). Based on the expression of CD8A and CXCL6, respectively, the high- and low-risk subgroups were divided. As shown, the higher the TIDE, the worse the reaction to immunotherapy (Figure 7D). In addition, we found that IC_50_ of veliparib, all-trans retinoic acid, NVP-AUY922, axitinib, and olaparib was higher in the high-risk subgroup except AKT inhibitor VIII (Figure 7E). In conclusion, the low-risk subgroup had a higher possibility of benefiting from immunotherapy and chemotherapy.

### Evaluation of CD8A and CXCL6 by RT-qPCR

**FIGURE 8:**
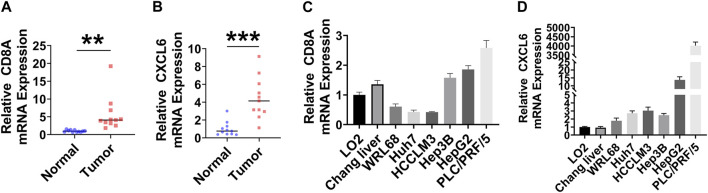
Evaluation of CD8A and CXCL6 by RT-qPCR. **(A, B)** mRNA expression levels of CD8A and CXCL6 in LIHC and adjacent normal samples (*n* = 11). **(C, D)** mRNA expression levels of CD8A and CXCL6 in normal human hepatic cell lines and human hepatoma cell lines (*n* = 6). **p < 0.01; ***p < 0.001.

mRNA expression levels of CD8A and CXCL6 were compared through RT-qPCR. As shown in Figures 8A–D, the higher positive expression of CD8A and CXCL6 was found in both LIHC tissues and most human liver cancer cell lines.

## Discussion

As a newly discovered programmed cell death mode, PANoptosis participates in fine-tuning anti-tumor immunity in TME by regulating the enrichment of immune cells and accelerating the death of tumor cells (Hao et al., 2022; Liu et al., 2022). Only a few malignant tumors in advanced LIHC can be completely removed by surgery. Furthermore, at present, safe and effective treatment for advanced LIHC can hardly be found, which leads to the rapid development and metastasis of the disease and the rising mortality (Llovet et al., 2021). Characterized by the infiltration of a large number of immune cells, LIHC is a highly immunogenic malignant tumor. Nowadays, even if the survival and immune status of LIHC patients have been constructed on the grounds of various forms of gene sets related to cell death, more research studies on the function of PANoptosis in LIHC are needed to be conducted. Multi-omics research refers to the simultaneous analysis of multiple types of biological molecules, such as genomics, transcriptomics, proteomics, and metabolomics. These omics types provide in-depth insights into different levels of biological systems, which can help provide a more comprehensive understanding of biological phenomena and aid in the diagnosis and treatment of diseases (Su et al., 2020).

The majority of the 19 PRGs discussed in this paper were related to the occurrence and development of LIHC. It has been demonstrated in the previous study that the expression of RIPK1, RIPK3, and MLKL-p has relevance to the better OS of patients with LIHC (Nicolè et al., 2022). Since the overexpression of PARP1 in patients with LIHC partly affected the poor clinical prognosis, sorafenib’s ability to eliminate residual tumors was strengthened with the help of the PARP inhibitor olaparib so that the treatment efficiency was improved (Yang et al., 2021). GSDMD-N and GSDMD, whose positive expression indicated poor prognosis, were upregulated in LIHC tissues or metastatic tissues (Lv et al., 2022). The results presented previously were entirely consistent with our research about differential expression of PRGs between LIHC and adjacent normal samples. Considering the relevance of LIHC classification to PRG expression, it was found in this paper that the prognosis of PRG cluster A showed a more positive result than that of PRG cluster B with its enrichment of immune-related pathways and immune cell infiltration. The correlational research suggested that RIPK3 was downregulated in LIHC-related macrophages, which indicated its connection with tumorigenesis, enhanced M2 polarization, and accumulation in TAM (Wu et al., 2020). Moreover, in LIHC cells with intrinsic RIPK3 deficiency, the knockout of MLKL is capable of enhancing the blocking of the immune checkpoint. Infiltrating CD8^+^ T cells could cause a better prognosis in LIHC, with RIPK1, RIPK3, and MLKL-p significantly correlated with CD3^+^ and CD8^+^ T cells in tumor (Nicolè et al., 2022; Jiang et al., 2023). In addition, inhibition of cellular FADD-like interleukin-1β-converting enzyme-inhibitory protein (c-FLIP) expression in LIHC cells could promote γδ T-cell-induced LIHC cell lysis (Chen et al., 2020). The larger part of GSDM members were considered to be positively related to the infiltration of B cells, neutrophils, and dendritic cells in LIHC (Hu et al., 2021). Correspondingly, the DEGs of two PRG clusters were enriched in tumor immune-related pathways, and two DEG clusters based on DEGs differed significantly in survival and PRG expression.

PRDEGs based on DEGs were screened to construct prognosis and calculate the risk score, and finally, CD8A and CXCL6 were selected. There were previous studies establishing that both genes were associated with LIHC. For instance, higher CLCF1–CXCL6/TGF-β axis levels were found to be related to poor prognosis in LIHC (Song et al., 2021). In addition, the low expression of CD8A in tumor tissue could predict the low survival rate of patients with LIHC (Tan et al., 2021). Thus, it was indicated that both PRDEGs could be used as underlying biomarkers for tumor diagnosis and treatment. On the basis of the risk score of each patient, patients were divided into low-risk and high-risk subgroups. The prognosis of the low-risk group was better, which was verified by the training and test queues. The study investigating the relationship between immune subtypes and the prognosis of LIHC suggested that the higher the expression of CD8A, the longer the survival time of the patients (Xu et al., 2020). These further implied the accuracy of the risk model.

The connection between risk score and the abundance of immune cells was investigated, and seven were positively correlated while five were negatively correlated. CD8A and CXCL6, two PRDEGs that were closely related to immune cells, have been found to be new indicators of the prognosis and immunotherapy response of other tumors. In bladder cancer, as a new protective gene, *CD8A* presented the highest correlation with T cells and M1 macrophages. Moreover, in advanced cancer patients receiving immunotherapy, it is noted that low CD8A expression showed an association with poor immunotherapy effect and survival (Zheng et al., 2022). Regarding adamantinomatous craniopharyngioma, the infiltrating abundance of γδ T cells was positively correlated with CXCL6, while CD8^+^ T cells were negatively correlated with CXCL6 (Lin et al., 2022). The analysis of 81 samples from LIHC patients revealed a significant correlation between the expression of CD8A and B cells (Garnelo et al., 2017). The findings were in line with the majority of our research results, suggesting the predictive potential of both PRDEGs. The effect of anti-tumor therapy in the low-risk subgroup turned out to be better than that of the high-risk subgroup, with its immune score and stromal score higher than those of the high-risk subgroup. A number of immune checkpoint gene expression differences between risk subgroups presented high expression in the low-risk subgroup, such as BTLA, CD160, CD244, CD274 (PD-L1), and PDCD1. By comparing the expression differences of PRGs in various risk subgroups, it was pointed out that the expression of NLRP3, MLKL, IRF1, AIM2, ZBP1, CASP1, and RIPK3 was higher in the low-risk subgroup. The relevant studies have reported that NLRP3 inflammasome upregulates the expression of PD-L1 in patients with diffuse large B-cell lymphoma (Lu et al., 2021), and IRF1 promoted the expression of PD-L1 in hepatoma cells (Xiao et al., 2019). Meanwhile, the TIDE score suggested a lower possibility of immune escape in the low-risk subgroup and a higher possibility of benefiting from the therapy of immune checkpoint inhibitors in the LIHC patients, which turned out to be consistent. The results of IC_50_ showed that the low-risk subgroup was more sensitive to chemotherapy drugs, and both immunotherapy and chemotherapy effects echoed with TME analysis. Therefore, all our results could be applied to guide clinical immunotherapy and chemotherapy for LIHC patients and contribute to a further understanding of the impact of PANoptosis on LIHC. Through the experiment, it was found out that CD8A and CXCL6 showed higher positive expression in both LIHC tissues and most human liver cancer cell lines, which implied that both PRDEGs might be potential biomarkers for the diagnosis and treatment of LIHC.

Surely, some limitations existed in the research. First and foremost, the data to be analyzed were from the public database, with few random prospective samples included. Second, the limited molecular biology experiments only achieved preliminary verification. However, relevant functional experiments have not been carried out. Finally, the inclusion of clinical features was not rich enough; thus, actual clinical cases were needed to evaluate our findings. In a nutshell, with the help of the combination of bioinformatics and molecular biology, it was suggested that PANoptosis was bound up with LIHC-related survival and immunity, and two PRDEGs were identified as potential markers for diagnosis and treatment. In this way, the study provided us with a richer understanding of PANoptosis in LIHC and a few strategies in the clinical therapy of LIHC.

## Data Availability

The original contributions presented in the study are included in the article/Supplementary Material, further inquiries can be directed to the corresponding authors.

## References

[B1] Banoth B. Tuladhar S. Karki R. Sharma B. R. Briard B. Kesavardhana S. (2020). Zbp1 promotes fungi-induced inflammasome activation and pyroptosis, apoptosis, and necroptosis (panoptosis). J. Biol. Chem. 295 (52), 18276–18283. Epub 2020/10/29. 10.1074/jbc.RA120.015924 33109609 PMC7939383

[B2] Beaufrère A. Calderaro J. Paradis V. (2021). Combined hepatocellular-cholangiocarcinoma: An update. J. hepatology 74 (5), 1212–1224. 10.1016/j.jhep.2021.01.035 33545267

[B3] Bray F. Ferlay J. Soerjomataram I. Siegel R. Torre L. Jemal A. (2018). Global cancer statistics 2018: Globocan estimates of incidence and mortality worldwide for 36 cancers in 185 countries. CA a cancer J. Clin. 68 (6), 394–424. 10.3322/caac.21492 30207593

[B4] Briard B. Malireddi R. Kanneganti T. (2021). Role of inflammasomes/pyroptosis and panoptosis during fungal infection. PLoS Pathog. 17 (3), e1009358. 10.1371/journal.ppat.1009358 33735255 PMC7971547

[B5] Chen J. Wang H. Zhou L. Liu Z. Chen H. Tan X. (2022). A necroptosis-related gene signature for predicting prognosis, immune landscape, and drug sensitivity in hepatocellular carcinoma. Cancer Med. 11 (24), 5079–5096. Epub 2022/05/14. 10.1002/cam4.4812 35560794 PMC9761093

[B6] Chen Z. Zheng Z. Feng L. Huo Z. Huang L. Fu M. (2020). Overexpression of mir-382 sensitizes hepatocellular carcinoma cells to γδ T cells by inhibiting the expression of C-flip. Mol. Ther. oncolytics 18, 467–475. 10.1016/j.omto.2020.07.012 32953981 PMC7479278

[B7] Chen Z. Zou Y. Zhang Y. Chen Z. Wu F. Jin H. (2022). A pyroptosis-based prognostic model for immune microenvironment estimation of hepatocellular carcinoma. Dis. markers 2022, 8109771. Epub 2022/01/21. 10.1155/2022/8109771 35047095 PMC8763514

[B8] Chung S. Kim K. Seong J. (2022). Biomarkers for locally advanced hepatocellular carcinoma patients treated with liver-directed combined radiotherapy. Liver cancer 11 (3), 247–255. 10.1159/000522000 35949293 PMC9218622

[B9] El-Khoueiry A. Sangro B. Yau T. Crocenzi T. Kudo M. Hsu C. (2017). Nivolumab in patients with advanced hepatocellular carcinoma (checkmate 040): An open-label, non-comparative, phase 1/2 dose escalation and expansion trial. Lancet (London, Engl. 389 (10088), 2492–2502. 10.1016/s0140-6736(17)31046-2 PMC7539326 28434648

[B10] Galluzzi L. Vitale I. Aaronson S. A. Abrams J. M. Adam D. Agostinis P. (2018). Molecular mechanisms of cell death: Recommendations of the nomenclature committee on cell death 2018. Cell death Differ. 25 (3), 486–541. Epub 2018/01/25. 10.1038/s41418-017-0012-4 29362479 PMC5864239

[B11] Garnelo M. Tan A. Her Z. Yeong J. Lim C. Chen J. (2017). Interaction between tumour-infiltrating B cells and T cells controls the progression of hepatocellular carcinoma. Gut 66 (2), 342–351. 10.1136/gutjnl-2015-310814 26669617 PMC5284473

[B12] Hao Y. Yang B. Yang J. Shi X. Yang X. Zhang D. (2022). Zbp1: A powerful innate immune sensor and double-edged sword in host immunity. Int. J. Mol. Sci. 23 (18), 10224. 10.3390/ijms231810224 36142136 PMC9499459

[B13] Hu K. Xu Z. Yao L. Yan Y. Zhou L. Li J. (2021). Integrated analysis of expression, prognostic value and immune infiltration of gsdms in hepatocellular carcinoma. Aging 13 (21), 24117–24135. 10.18632/aging.203669 34731088 PMC8610125

[B14] Jiang M. Qi L. Li L. Wu Y. Song D. Li Y. (2021). Caspase-8: A key protein of cross-talk signal way in "panoptosis" in cancer. Int. J. cancer 149 (7), 1408–1420. 10.1002/ijc.33698 34028029

[B15] Jiang X. Deng W. Tao S. Tang Z. Chen Y. Tian M. (2023). A ripk3-independent role of mlkl in suppressing parthanatos promotes immune evasion in hepatocellular carcinoma. Cell Discov. 9 (1), 7. 10.1038/s41421-022-00504-0 36650126 PMC9845215

[B16] Karim M. Singal A. Kum H. Lee Y. Park S. Rich N. (2022). Clinical characteristics and outcomes of nonalcoholic fatty liver disease-associated hepatocellular carcinoma in the United States. Clin. gastroenterology hepatology official Clin. Pract. J. Am. Gastroenterological Assoc. 21, 670–680. e18. 10.1016/j.cgh.2022.03.010 PMC9481743 35307595

[B17] Karki R. Sharma B. R. Lee E. Banoth B. Malireddi R. K. S. Samir P. (2020). Interferon regulatory factor 1 regulates panoptosis to prevent colorectal cancer. JCI insight 5 (12), e136720. Epub 2020/06/20. 10.1172/jci.insight.136720 32554929 PMC7406299

[B18] Karki R. Sharma B. R. Tuladhar S. Williams E. P. Zalduondo L. Samir P. (2021). Synergism of TNF-α and IFN-γ triggers inflammatory cell death, tissue damage, and mortality in SARS-CoV-2 infection and cytokine shock syndromes. Cell 184 (1), 149–168.e17. e17. Epub 2020/12/06. 10.1016/j.cell.2020.11.025 33278357 PMC7674074

[B19] Karki R. Sundaram B. Sharma B. R. Lee S. Malireddi R. K. S. Nguyen L. N. (2021). Adar1 restricts zbp1-mediated immune response and panoptosis to promote tumorigenesis. Cell Rep. 37 (3), 109858. Epub 2021/10/24. 10.1016/j.celrep.2021.109858 34686350 PMC8853634

[B20] Kesavardhana S. Malireddi R. K. S. Kanneganti T. D. (2020). Caspases in cell death, inflammation, and pyroptosis. Annu. Rev. Immunol. 38, 567–595. Epub 2020/02/06. 10.1146/annurev-immunol-073119-095439 32017655 PMC7190443

[B21] Lee S. Karki R. Wang Y. Nguyen L. Kalathur R. Kanneganti T. (2021). Aim2 forms a complex with pyrin and Zbp1 to drive panoptosis and host defence. Nature 597 (7876), 415–419. 10.1038/s41586-021-03875-8 34471287 PMC8603942

[B22] Lin D. Zhao W. Yang J. Wang H. Zhang H. (2022). Integrative analysis of biomarkers and mechanisms in adamantinomatous craniopharyngioma. Front. Genet. 13, 830793. 10.3389/fgene.2022.830793 35432485 PMC9006448

[B23] Liu J. Hong M. Li Y. Chen D. Wu Y. Hu Y. (2022). Programmed cell death tunes tumor immunity. Front. Immunol. 13, 847345. 10.3389/fimmu.2022.847345 35432318 PMC9005769

[B24] Llovet J. Kelley R. Villanueva A. Singal A. Pikarsky E. Roayaie S. (2021). Hepatocellular carcinoma. Nat. Rev. Dis. Prim. 7 (1), 6. 10.1038/s41572-020-00240-3 33479224 PMC13537433

[B25] Lu F. Zhao Y. Pang Y. Ji M. Sun Y. Wang H. (2021). Nlrp3 inflammasome upregulates Pd-L1 expression and contributes to immune suppression in lymphoma. Cancer Lett. 497, 178–189. 10.1016/j.canlet.2020.10.024 33091534

[B26] Lv T. Xiong X. Yan W. Liu M. Xu H. He Q. (2022). Targeting of gsdmd sensitizes hcc to anti-Pd-1 by activating cgas pathway and downregulating Pd-L1 expression. J. Immunother. cancer 10 (6), e004763. 10.1136/jitc-2022-004763 35688553 PMC9189836

[B27] Malireddi R. Kesavardhana S. Kanneganti T. (2019). Zbp1 and Tak1: Master regulators of Nlrp3 inflammasome/pyroptosis, apoptosis, and necroptosis (Pan-Optosis). Front. Cell. Infect. Microbiol. 9, 406. 10.3389/fcimb.2019.00406 31850239 PMC6902032

[B28] Malireddi R. K. S. Karki R. Sundaram B. Kancharana B. Lee S. Samir P. (2021). Inflammatory cell death, panoptosis, mediated by cytokines in diverse cancer lineages inhibits tumor growth. ImmunoHorizons 5 (7), 568–580. Epub 2021/07/23. 10.4049/immunohorizons.2100059 34290111 PMC8522052

[B29] Malireddi R. K. S. Kesavardhana S. Karki R. Kancharana B. Burton A. R. Kanneganti T. D. (2020). Ripk1 distinctly regulates yersinia-induced inflammatory cell death, panoptosis. ImmunoHorizons 4 (12), 789–796. Epub 2020/12/15. 10.4049/immunohorizons.2000097 33310881 PMC7906112

[B30] Malireddi R. K. S. Tweedell R. E. Kanneganti T. D. (2020). Panoptosis components, regulation, and implications. Aging 12 (12), 11163–11164. Epub 2020/06/24. 10.18632/aging.103528 32575071 PMC7343493

[B31] Newton K. Wickliffe K. E. Maltzman A. Dugger D. L. Reja R. Zhang Y. (2019). Activity of caspase-8 determines plasticity between cell death pathways. Nature 575 (7784), 679–682. Epub 2019/11/15. 10.1038/s41586-019-1752-8 31723262

[B32] Nguyen L. Kanneganti T. (2022). Panoptosis in viral infection: The missing puzzle piece in the cell death field. J. Mol. Biol. 434 (4), 167249. 10.1016/j.jmb.2021.167249 34537233 PMC8444475

[B33] Nicolè L. Sanavia T. Cappellesso R. Maffeis V. Akiba J. Kawahara A. (2022). Necroptosis-driving genes *RIPK1, RIPK3* and *MLKL-p* are associated with intratumoral CD3^+^ and CD8^+^ T cell density and predict prognosis in hepatocellular carcinoma. J. Immunother. cancer 10 (3), e004031. 10.1136/jitc-2021-004031 35264437 PMC8915343

[B34] Place D. Lee S. Kanneganti T. (2021). Panoptosis in microbial infection. Curr. Opin. Microbiol. 59, 42–49. 10.1016/j.mib.2020.07.012 32829024 PMC7438227

[B35] Samir P. Malireddi R. Kanneganti T. (2020). The panoptosome: A deadly protein complex driving pyroptosis, apoptosis, and necroptosis (panoptosis). Front. Cell. Infect. Microbiol. 10, 238. 10.3389/fcimb.2020.00238 32582562 PMC7283380

[B36] Song M. He J. Pan Q. Yang J. Zhao J. Zhang Y. (2021). Cancer-associated fibroblast-mediated cellular crosstalk supports hepatocellular carcinoma progression. Hepatol. Baltim. Md) 73 (5), 1717–1735. 10.1002/hep.31792 33682185

[B37] Su J. Song Q. Qasem S. O'Neill S. Lee J. Furdui C. (2020). Multi-omics analysis of brain metastasis outcomes following craniotomy. Front. Oncol. 10, 615472. 10.3389/fonc.2020.615472 33889540 PMC8056216

[B38] Tan H. Wang N. Zhang C. Chan Y. Yuen M. Feng Y. (2021). Lysyl oxidase-like 4 fosters an immunosuppressive microenvironment during hepatocarcinogenesis. Hepatol. Baltim. Md) 73 (6), 2326–2341. 10.1002/hep.31600 PMC8251926 33068461

[B39] Wang Y. Kanneganti T. D. (2021). From pyroptosis, apoptosis and necroptosis to panoptosis: A mechanistic compendium of programmed cell death pathways. Comput. Struct. Biotechnol. J. 19, 4641–4657. Epub 2021/09/11. 10.1016/j.csbj.2021.07.038 34504660 PMC8405902

[B40] Wu L. Zhang X. Zheng L. Zhao H. Yan G. Zhang Q. (2020). Ripk3 orchestrates fatty acid metabolism in tumor-associated macrophages and hepatocarcinogenesis. Cancer Immunol. Res. 8 (5), 710–721. 10.1158/2326-6066.cir-19-0261 32122992

[B41] Xiao G. Jin L. Liu C. Wang Y. Meng Y. Zhou Z. (2019). Ezh2 negatively regulates Pd-L1 expression in hepatocellular carcinoma. J. Immunother. cancer 7 (1), 300. 10.1186/s40425-019-0784-9 31727135 PMC6854886

[B42] Xu D. Liu X. Wang Y. Zhou K. Wu J. Chen J. (2020). Identification of immune subtypes and prognosis of hepatocellular carcinoma based on immune checkpoint gene expression profile. Biomed. Pharmacother. = Biomedecine Pharmacother. 126, 109903. 10.1016/j.biopha.2020.109903 32113055

[B43] Yang X. Kong F. Qi L. Lin J. Yan Q. Loong J. (2021). Parp inhibitor Olaparib overcomes Sorafenib resistance through reshaping the pluripotent transcriptome in hepatocellular carcinoma. Mol. cancer 20 (1), 20. 10.1186/s12943-021-01315-9 33485358 PMC7824946

[B44] Zheng M. Karki R. Vogel P. Kanneganti T. D. (2020). Caspase-6 is a key regulator of innate immunity, inflammasome activation, and host defense. Cell 181 (3), 674–687. e13. Epub 2020/04/17. 10.1016/j.cell.2020.03.040 32298652 PMC7425208

[B45] Zheng Z. Guo Y. Huang X. Liu J. Wang R. Qiu X. (2022). Cd8a as a prognostic and immunotherapy predictive biomarker can Be evaluated by mri radiomics features in bladder cancer. Cancers 14 (19), 4866. 10.3390/cancers14194866 36230788 PMC9564077

[B46] Zhu A. Finn R. Edeline J. Cattan S. Ogasawara S. Palmer D. (2018). Pembrolizumab in patients with advanced hepatocellular carcinoma previously treated with Sorafenib (Keynote-224): A non-randomised, open-label phase 2 trial. Lancet Oncol. 19 (7), 940–952. 10.1016/s1470-2045(18)30351-6 29875066

[B47] Zhu J. Tang B. Lv X. Meng M. Weng Q. Zhang N. (2020). Identifying apoptosis-related transcriptomic aberrations and revealing clinical relevance as diagnostic and prognostic biomarker in hepatocellular carcinoma. Front. Oncol. 10, 519180. Epub 2021/03/09. 10.3389/fonc.2020.519180 33680905 PMC7931692

