# Supplementary material for: Identification of PANoptosis-relevant subgroups to evaluate the prognosis and immune landscape of patients with liver hepatocellular carcinoma
Source: Front Cell Dev Biol. 2023 May 30;11:1210456. doi: 10.3389/fcell.2023.1210456 (PMC10267832; doi:10.3389/fcell.2023.1210456)
Supplement: Supplementary file 2 [file Table1.DOCX]

Supplementary Material

**Identification of PANoptosis-relevant subgroups to evaluate prognosis and immune landscape of patients with liver hepatocellular carcinoma**

Zhengwei Zhang 1,2†, Feng Zhang 1,2†, Ping Pang 1, Yapeng Li 1,2, Xiaoning Chen 2, Shibo Sun 2*, Yu Bian 1*

**Supplementary Figure S1** Unsupervised clustering of PANoptosis-related genes and Consensus matrix heatmaps for *k* = 1-9.

**Supplementary Figure S2** KM curves of the relationship between PRGs expression and the prognosis of LIHC patients based on TCGA database (*p* < 0.05).

**Supplementary Figure S3** DEGs clustering and Consensus matrix heatmaps for *k* = 1-9.

**Supplementary Figure S4** Validation of the risk score. (A) Expression of 2 genes in two risk subgroups from the test queue. (B) Risk score and survival outcome of each patient from the test queue. (C) The better prognosis from low-risk subgroup in the test queue (*p* = 0.009). (D) The AUCs of 1, 3, 5-years survival period were 0.577, 0.632 and 0.673 in the test queue, respectively. (E) Expression of 2 genes in two risk subgroups from the training queue. (F) Risk score and survival outcome of each patient from the training queue. (G) The better prognosis from low-risk subgroup in the training queue (*p* < 0.001). (H) The AUCs of 1, 3, 5-years survival period were 0.693, 0.704 and 0.750 in the training queue, respectively.

**Supplementary Figure S5** Validation of survival analysis for key genes. (A) CD8A, with *p* value of 0.057. (B) CXCL6, with *p* value of 0.042, was a prognostic factor for hepatocellular carcinoma.

**Supplementary Table S1** definite clinical information for LIHC patients.

**Supplementary Table S2** 19 PRGs according to previous researches.

**Supplementary Table S3** the primers for RT-qPCR.

**Supplementary Table S4** the raw data from RT-qPCR.
