# Supplementary figures and images for: Identification of PANoptosis-relevant subgroups to evaluate the prognosis and immune landscape of patients with liver hepatocellular carcinoma
Source: Front Cell Dev Biol. 2023 May 30;11:1210456. doi: 10.3389/fcell.2023.1210456 (PMC10267832; doi:10.3389/fcell.2023.1210456)

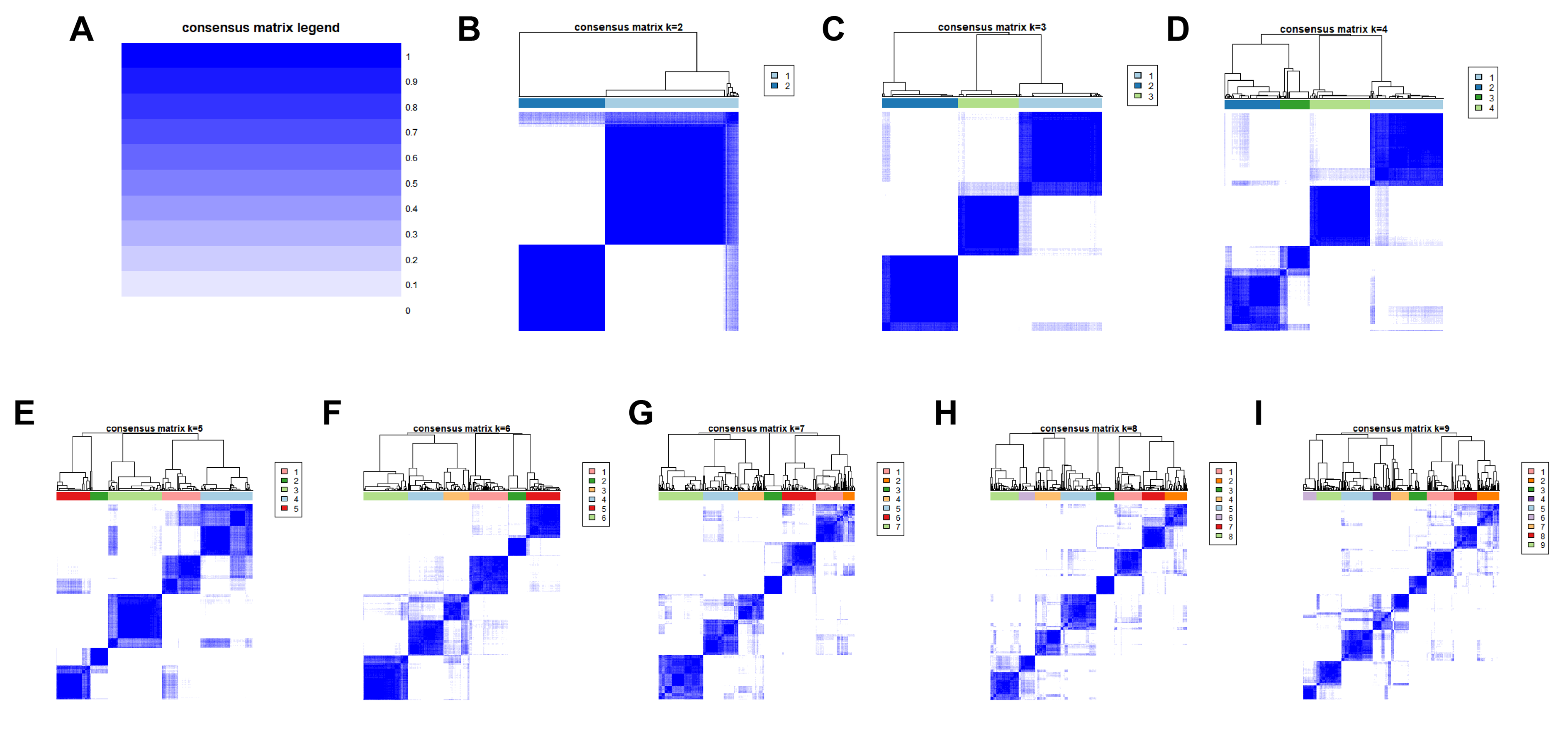

Supplement: Supplementary file 3 [file Image3.TIF]

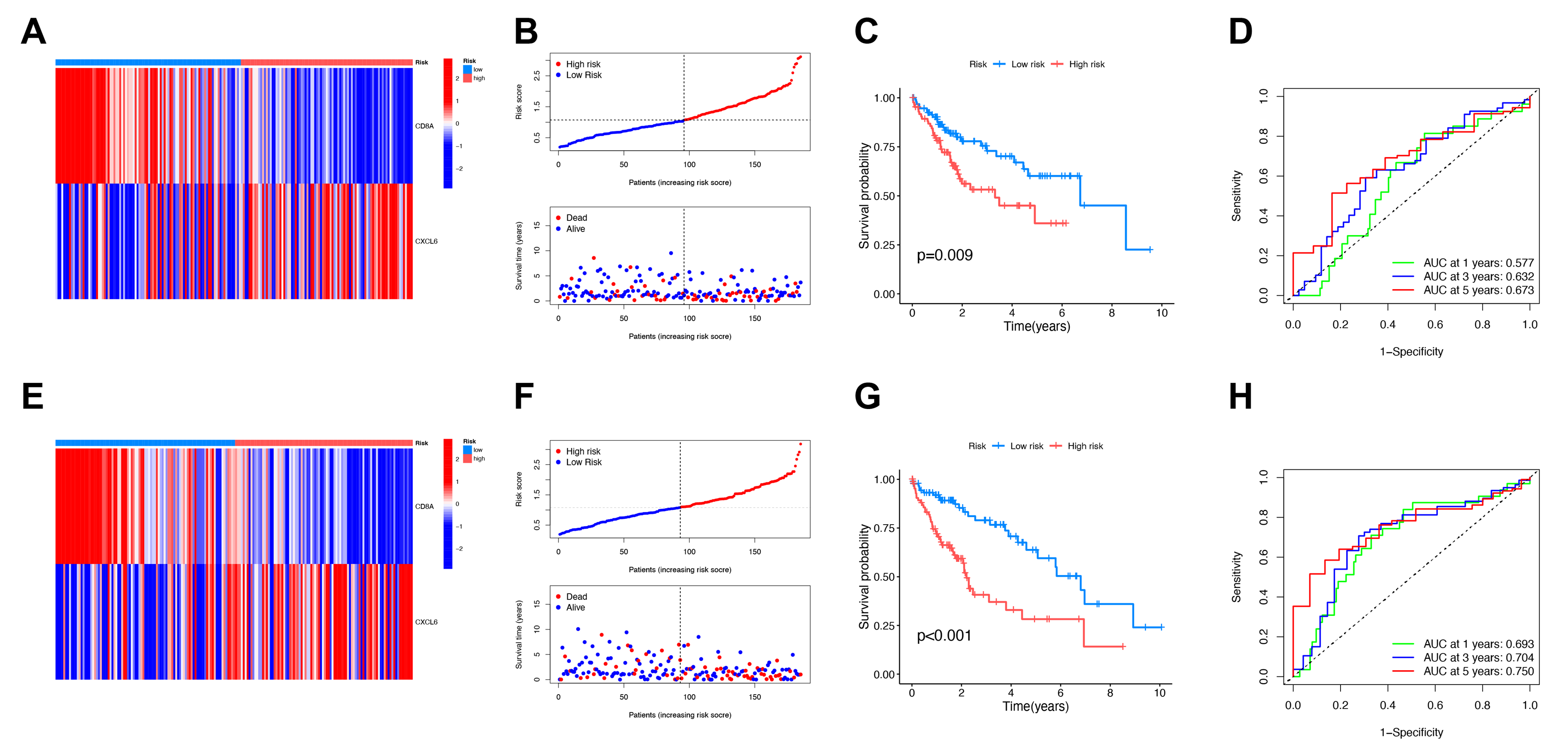

Supplement: Supplementary file 4 [file Image4.TIF]

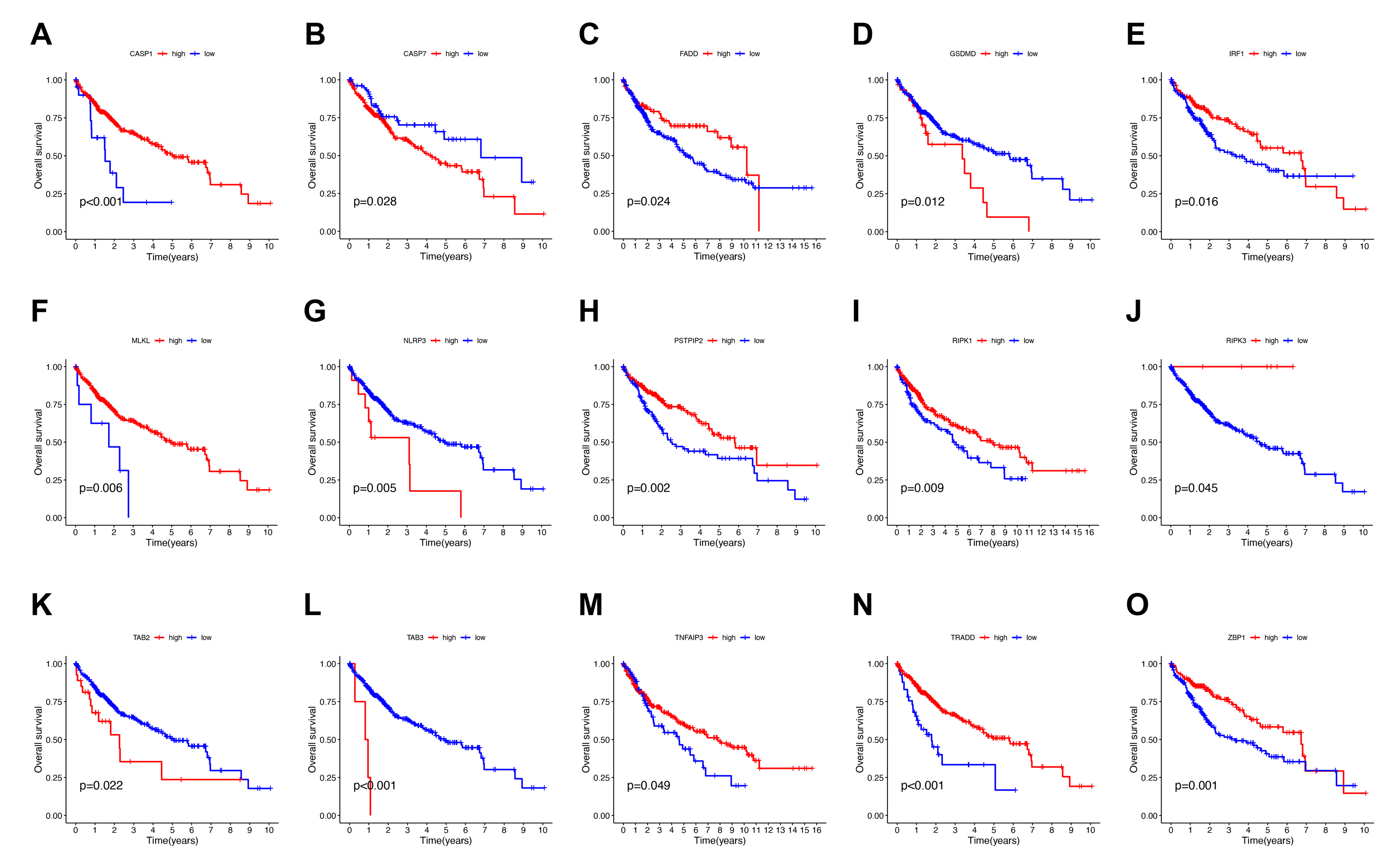

Supplement: Supplementary file 5 [file Image2.TIF]

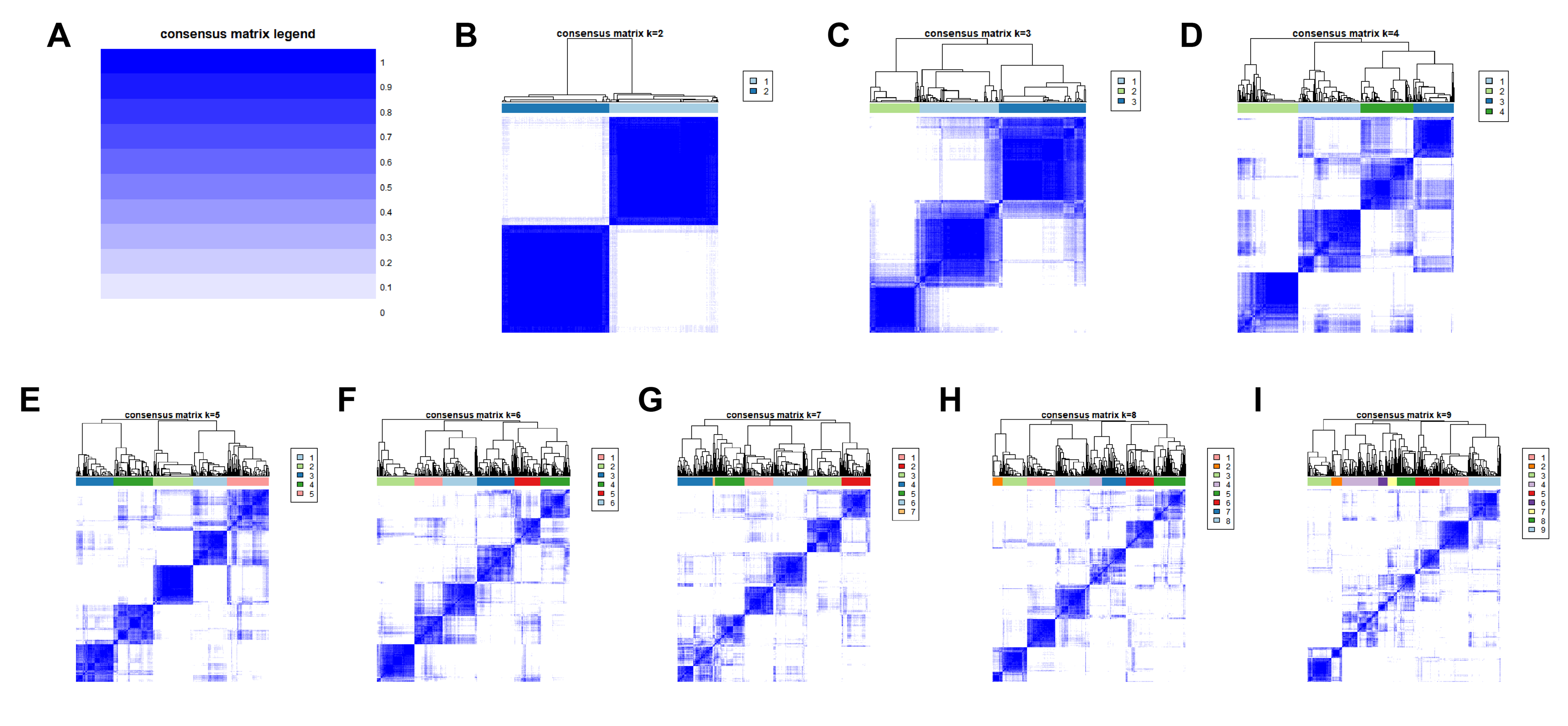

Supplement: Supplementary file 7 [file Image1.TIF]

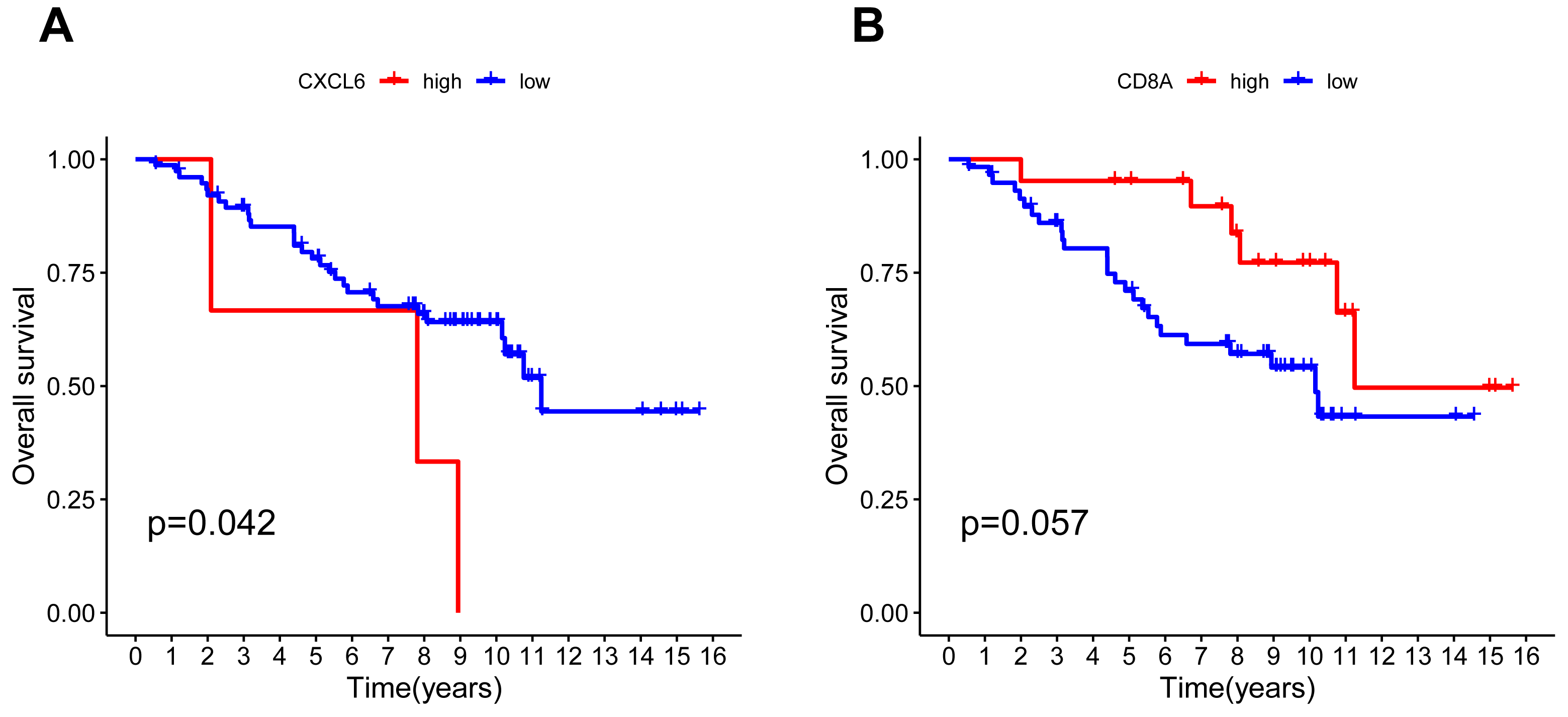

Supplement: Supplementary file 10 [file Image5.TIF]
