# Supplementary material for: Identification of PANoptosis-relevant subgroups to evaluate the prognosis and immune landscape of patients with liver hepatocellular carcinoma
Source: Front Cell Dev Biol. 2023 May 30;11:1210456. doi: 10.3389/fcell.2023.1210456 (PMC10267832; doi:10.3389/fcell.2023.1210456)
Supplement: Supplementary file 9 [file Table6.DOCX]

https://www.jianguoyun.com/p/DaDkhYkQ8KHMCxjGpYMFIAA
